# Spinal cord infarction after withdrawal of veno-arterial extracorporeal membrane oxygenation for cardiogenic shock: A case report

**DOI:** 10.1097/MD.0000000000031743

**Published:** 2022-11-11

**Authors:** Hideya Itagaki, Kohei Suzuki, Tomoya Oizumi, Keiko Nakagawa, Yoshinobu Abe, Tomoyuki Endo

**Affiliations:** a Division of Emergency and Disaster Medicine, Tohoku Medical and Pharmaceutical University Hospital, Miyagi, Japan; b Emergency Department, Tohoku Medical and Pharmaceutical University Hospital, Miyagi, Japan.

**Keywords:** cardiogenic shock, cardiopulmonary arrest, case report, spinal cord infarction

## Abstract

Spinal cord infarction is a rare central nervous system angiopathy that impairs motor, sensory, and autonomic nerves and occurs due to various reasons. This study reports a case of spinal cord infarction in a patient following myocardial infarction that was managed by veno-arterial extracorporeal membrane oxygenation (VA-ECMO).

**Case summary:**

A 78-year-old Japanese man visited the emergency department with a complaint of chest tightness. He had a history of hypertension, dyslipidemia, diabetes, chronic renal failure, and postoperative bladder cancer. Myocardial infarction was diagnosed after ST elevation in lead aVR was identified by electrocardiogram during the visit, and cardiopulmonary arrest occurred twice during our examination and treatment. After percutaneous coronary intervention with an intra-aortic balloon pump and VA-ECMO, the patient was admitted to the intensive care unit. His circulation stabilized, and he was withdrawn from the intra-aortic balloon pump on day 3 of illness and from VA-ECMO on day 4. However, his consciousness remained impaired. When the patient’s consciousness improved on day 14, lower limb weakness was identified. Magnetic resonance imaging conducted on the following day revealed spinal cord infarction in the 5^th^ to 12^th^ thoracic vertebrae.

**Conclusion:**

Spinal cord infarction due to VA-ECMO is extremely rare but has a poor neurological prognosis upon onset. Necessary countermeasures include conducting regular neurological examinations and high blood pressure maintenance, which is very difficult in VA-ECMO patients. Therefore, patient care will benefit from the experiences reported in such cases.

## 1. Introduction

Spinal cord infarction is a rare central nervous system angiopathy and a serious disease that causes motor paralysis, sensory disorders (e.g., decreased sensation for pain and temperature), and autonomic neuropathy.^[1]^ Long-term follow-up studies have reported lower mortality but worse functional outcomes in these patients compared with that noted in patients with cerebral infarction.^[2]^ Various factors, including arteriosclerosis, aortic lesions (regardless of surgery), degenerative spinal diseases, myocardial infarction, epidural anesthesia, and systemic hypoperfusion, cause spinal cord infarction.^[3]^ Although rare, veno-arterial extracorporeal membrane oxygenation (VA-ECMO) can lead to spinal cord infarction.

Herein, we report a case of spinal cord infarction due to VA-ECMO following withdrawal of VA-ECMO management for myocardial infarction.

## 2. Case presentation

The Tohoku Medical and Pharmaceutical University Ethics Committee approved this study (approval number: 2022-4-010). The patient and his family provided written informed consent for publication of this case report and accompanying images.

A 78-year-old Japanese man visited the emergency department for worsening chest tightness that had persisted for approximately 2 weeks. The patient had a history of hypertension, dyslipidemia, diabetes, chronic renal failure, and postoperative bladder cancer and was taking nicardipine, sitagliptin phosphate hydrate, atorvastatin, sodium hydrogen carbonate, and senna. At the initial visit, the patient’s vital signs were as follows: blood pressure, 116/92 mm Hg; pulse rate, 92 beats/minute; respiratory rate, 24 breaths/minute; body temperature, 36.4°C; and blood oxygen saturation (SpO_2_), 95% in room air. The electrocardiogram showed repeated ventricular tachycardia; however, other physical findings, including neurological findings, were normal.

We suspected acute coronary syndrome and thus conducted blood tests, echocardiography, and electrocardiography. Electrocardiography showed ST elevation in lead aVR and ST depression in leads I/II/aVF/V4–6. Echocardiography revealed a high degree of wall hypokinesia centered on the anterior septal region. During these tests, the patient sustained a cardiopulmonary arrest. After 8 minutes of resuscitation, the heartbeat returned, and the patient was moved to the catheter room for percutaneous coronary intervention (PCI). However, the patient experienced another cardiopulmonary arrest while preparations were underway; therefore, resuscitation was attempted again, and the heartbeat returned after 5 minutes.

Circulation was unstable even with vasopressor use (noradrenaline 0.4 µg/kg/minute); hence, an emergency coronary angiography was conducted after introducing an intra-aortic balloon pump (IABP) and VA-ECMO. Coronary angiography showed approximately 99–100% stenosis of the left main trunk; therefore, PCI was conducted at the same site. The activated clotting time (ACT) when the patient entered the catheter room was 137 seconds, and a heparin bolus was intravenously injected to increase the ACT to ≥ 250 seconds during PCI; the final ACT was 316 seconds. Although the patient entered the intensive care unit 1 hour after PCI, a blood test conducted immediately after intensive care unit entry showed abnormal coagulation [prothrombin time (PT)/international normalized ratio, 2.71; activated partial thromboplastin time (APTT), >200 seconds; D-dimer, 23.46 μg/mL]. Furthermore, bleeding from the nasogastric tube and severe anemia (hemoglobin, 6.4 g/dL) were confirmed. Thus, VA-ECMO was performed without heparin, and blood transfusion (red blood cells, 4 units; fresh frozen plasma, 4 units) was initiated.

The next morning (day 2 of hospitalization), the blood test results indicated that the coagulation parameters had improved (PT, 1.490; APTT, 67.9 sec; D-dimer, 7.99 μg/mL), but the patient was still anemic (hemoglobin, 8.6 g/dL) and thrombocytopenic (platelet count, 81,000/µL). However, afternoon blood tests indicated an elevated APTT (83.6 seconds); thus, another blood transfusion was performed (red blood cells, 8 units; fresh frozen plasma: 6 units; platelet concentrates, 20 units).

On day 3 of hospitalization, blood tests indicated that the coagulation ability had increased again (PT, 1.430; APTT, 78.2 seconds). Therefore, another 2 units of fresh frozen plasma were transfused. However, as the heart circulation recovered spontaneously, VA-ECMO was withdrawn on the same day.

Until day 3 of hospitalization, the patient had not woken up since admission. Consequently, the sedative (midazolam) was discontinued. On day 4 of hospitalization, we withdrew the patient from the IABP with stable circulation and discontinuation of the analgesic (fentanyl). Cranial computed tomography and electroencephalography were conducted on days 4 and 7 of hospitalization, respectively, but there were no obvious abnormalities. Thus, we suspected prolonged impaired consciousness after resuscitation.

**Figure 1.**
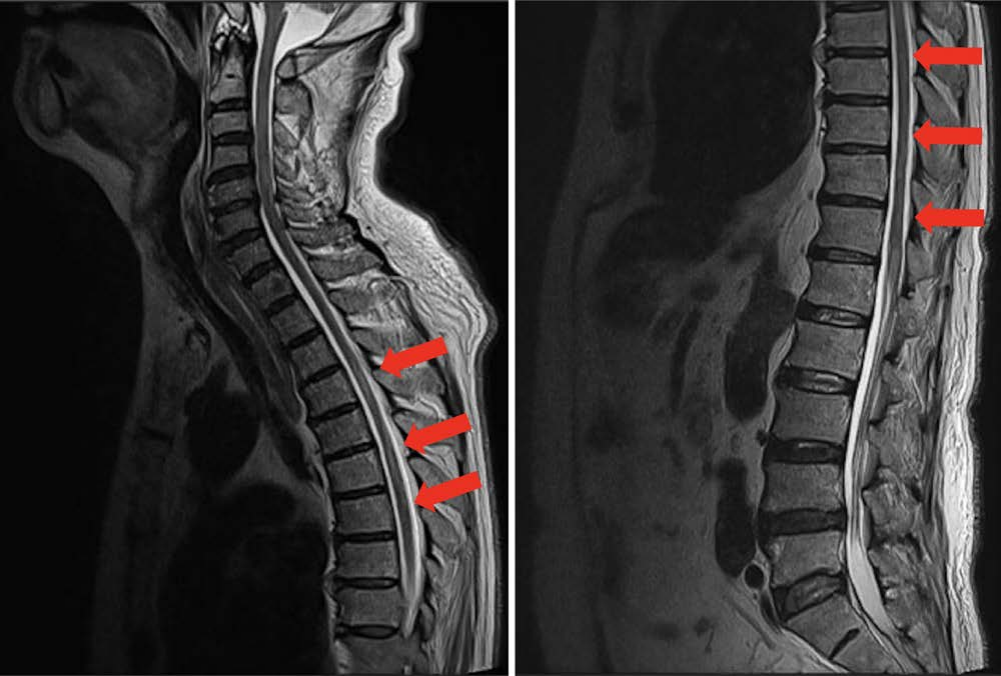
T2-weighted sagittal magnetic resonance image showing spinal cord infarction [red arrow].

The patient began to open his eyes to verbal stimulus on day 12 and could communicate on day 14 of hospitalization, which is when the ventilator support was withdrawn. Post-ventilator examination revealed that the patient had lost the ability to move his lower limbs. Spinal magnetic resonance imaging was conducted the next day (day 15), and T2-weighted signal hyperintensity was noted in the spinal cord from the fifth to the twelfth thoracic vertebrae, with spinal cord infarction (Fig. 1). Six months after discharge, the patient’s circulation was stable, but lower limb paralysis persisted; therefore, he is undergoing rehabilitation.

## 3. Discussion

Spinal cord infarction is a rare form of central nervous system angiopathy, accounting for approximately 1% of patients hospitalized for nervous system vascular lesions.^[1]^ Salvador et al reported that the most common causes of spinal cord infarction (excluding spinal disorders, tumors, and inflammatory lesions) were idiopathic (36.1%), followed by aortic surgery (25%), arteriosclerosis and embolism (19.4%), systemic hypoperfusion (e.g., cardiac arrest and hypovolemic shock) (11.1%), and aortic lesions without surgery (8.3%); however, spinal cord infarction has also been reported as a complication of PCI, IABP, and VA-ECMO.^[3–6]^ Spinal cord infarction due to VA-ECMO is very rare, with only few cases reported in the literature (Table 1). We suggest a causal relationship in our case as there were no neurological signs or symptoms before VA-ECMO. However, cardiac arrest occurred twice, and VA-ECMO, PCI, and IABP were all performed. Therefore, factors other than VA-ECMO must also be considered as potential causes of spinal cord infarction.

**Table 1:** Review of literature.

Author	Case No.	Age	Sex	VA-ECMO Reason	IABP	Cardiac arrest	Coronary angiography before SCI?	VA-ECMO Duration (days)	Days to the first signs of a neurological deficit or radiological diagnosis of SCI	Survived to hospital discharge?	Improvements in neurological findings?	Maintained an average blood pressure of 90 mm Hg
Loïc Le Guennec	Case 1	35	Male	Cocaine-induced cardiomyopathy	No	Yes	Yes	4	55	Yes	Yes	NA
	Case 2	48	Male	Pneumococcal pneumonia septic cardiomyopathy	No	Yes	Yes	29	59	Yes	No	NA
	Case 3	56	Male	Cardiogenic shock after AMI and cardiac arrest	No	Yes	Yes	2	3	Yes	No	NA
	Case 4	62	Male	Primary graft dysfunction after orthotopic heart transplantation	No	No	No	47	54	No	No	NA
	Case 5	43	Male	Cardiogenic shock after AMI	Yes	No	Yes	4	6	Yes	Yes	NA
	Case 6	62	Male	Ischaemic dilated cardiomyopathy and primary graft dysfunction after orthotopic heart transplantation	Yes	No	Yes	13	50	Yes	Yes	NA
Michael Salna	Case 7	29	Male	Heart-liver transplant complicated by graft dysfunction	NA	No	NA	12	14	NA	No	NA
	Case 8	59	Female	Cardiogenic shock	NA	No	NA	7	8	NA	No	NA
	Case 9	46	Female	Acute decompensated heart failure	NA	No	NA	12	12	NA	No	NA
	Case 10	56	Female	Primary graft dysfunction after orthotopic heart transplantation	NA	No	NA	25	25	NA	No	NA
Beomsu Shin	Case 11	81	Female	Cardiogenic shock after AMI and cardiac arrest	No	No	Yes	NA	1	Yes	No	No
Behnoosh Samadi	Case 12	37	Female	Viral cardiomyopathy	Yes	No	No	10	10	Yes	No	NA
	Case 13	43	Female	Cardiogenic shock after AMI and cardiotomy	Yes	Yes	Yes	9	7	Yes	Yes	NA
	Case 14	19	Female	Peripartum cardiomyopathy	Yes	Yes	No	3	10	NR	No	NA
Peter Magnusson	Case 15	28	Female	Perimyocarditis	No	Yes	No	21	NR	Yes	No	NA
Shivanand Gangahanumaiah	Case 16	49	Female	Primary graft dysfunction after orthotopic heart transplantation	No	No	No	10	24	No	No	NA
	Case 17	73	Male	Post-cardiotomy cardiogenic shock	No	No	No	10	12	No	No	NA
	Case 18	38	Female	Primary graft dysfunction after orthotopic heart transplantation	No	No	No	13	70	No	No	NA
Takeshi Oda	Case 19	6	Male	H1N1 influenza myocarditis	No	No	No	4.5	7.5	Yes	No	NA
Our hospital	Case 20	78	Male	Cardiogenic shock after AMI and cardiac arrest	Yes	Yes	Yes	3	15	Yes	No	No

IABP = intra-aortic balloon pump, VA-ECMO = veno-arterial extracorporeal membrane oxygenation.

**Figure 2.**
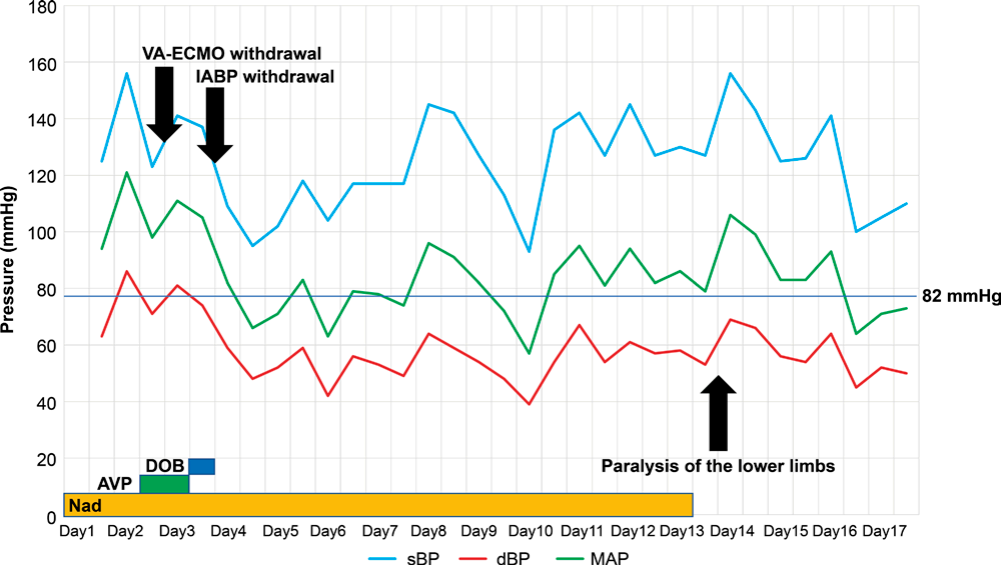
Blood pressure trend and events. dBP = diastolic blood pressure, MAP = mean arterial pressure, sBP = systolic blood pressure.

Cardiac arrest and systemic hypoperfusion also cause spinal cord infarction. Longer cardiac arrest times are associated with a higher frequency of spinal cord injury, with 61% of cases exhibiting spinal cord injuries when the arrest duration exceeds 20 minutes; however, if the duration is less than 10 minutes, the injury rate is approximately 33%.^[7]^ In our case, resuscitation was performed for less than 10 minutes on both occasions (5 and 8 minutes). We also considered systemic hypoperfusion, but the systolic blood pressure was ≥ 121 mm Hg, and the mean blood pressure was ≥ 82 mm Hg until day 15 of illness when the spinal cord infarction diagnosis was made. Thus, the patient was not in a state of hypoperfusion during hospitalization (Fig. 2).

**Figure 3.**
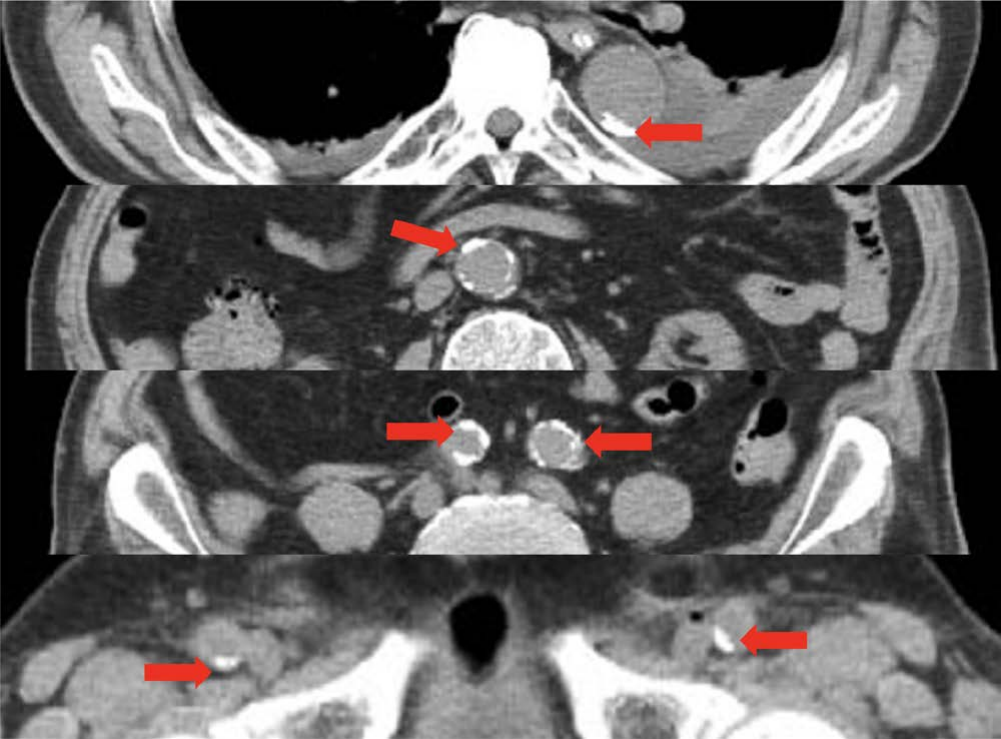
Calcification [red arrow] from the descending aorta to the common iliac artery to the femoral artery.

PCI can potentially cause spinal cord infarction, believed to result from the reduction in perfusion pressure of the internal iliac artery to an extreme degree due to blood flow disturbances caused by the catheter reaching the aorta via the common iliac artery of the femoral artery, thereby reducing the blood supply to the spinal cord. Another possibility is that operating a catheter in a blood vessel where arteriosclerosis is progressing causes the plaque to rupture, leading to embolism. In our case, we identified calcification from the descending aorta to the common iliac artery to the femoral artery (Fig. 3). Therefore, the possibility that a plaque was destroyed due to operating of the catheter cannot be discounted.^[4–6]^ However, catheterization was performed for IABP and extracorporeal membrane oxygenation (ECMO), meaning that the infarction in our case was likely not PCI-specific.

**Figure 4.**
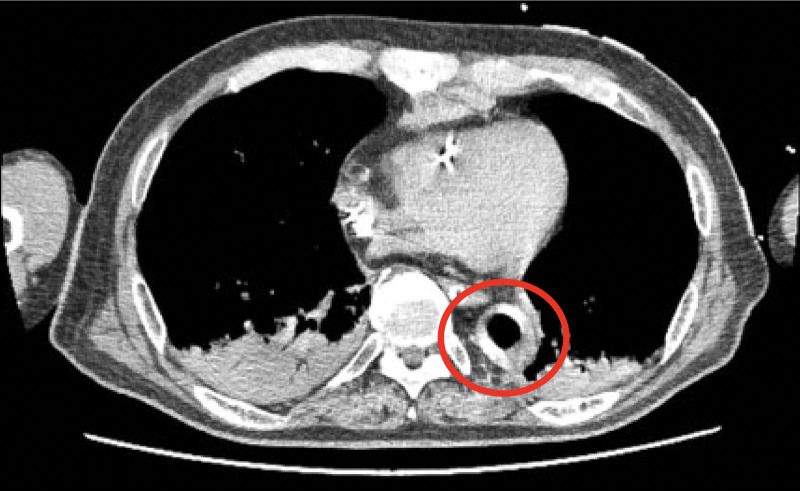
Difference between balloon diameter (16 mm) and descending aortic diameter (26 mm) [red circle].

IABP is hypothesized to cause spinal cord infarction by dissecting an aortic hematoma due to direct trauma from the balloon, branch artery vasospasms, and ischemia from aortic blockage due to balloon dilation.^[8]^ Behnoosh et al reported 3 cases citing this mechanism, wherein the descending aorta diameters were 12 to 14 mm in these cases, but the balloon diameter was 16 mm. Therefore, at 1 instance the balloon completely occluded the blood vessel. We also placed a 16-mm diameter balloon, but the descending aorta was 26 mm in diameter. Thus, the distance to the wall was sufficient (Fig. 4), and blockage or continuous contact with the aortic wall was unlikely to occur.

Cardiac arrest, systemic hypoperfusion, PCI, and IABP are all suspected causes of spinal cord infarction, but we should not conclude that they must be the leading causes of spinal cord infarction; VA-ECMO-induced spinal cord infarction should also be considered. Spinal cord infarction due to VA-ECMO is rare, but a possible reason is “mixing point” turbulence, wherein the heartbeat and the retrograde blood flow due to VA-ECMO collide, promoting platelet aggregation and thrombus formation.^[9]^ Rastan et al conducted autopsies on 78 individuals who died after circulatory support by ECMO following cardiotomy, reporting a breakdown of postoperative complications that were not clinically recognized. They identified 25 cases of venous thrombosis and 24 cases of systemic thromboembolism, with a high incidence of thrombosis.^[10]^ Anticoagulant therapy is administered to prevent thrombus and embolism, usually by intravenous heparin. The target treatment range for ECMO is 60–80 seconds for standard bleeding risk cases and 40 to 60 seconds for high bleeding risk cases.^[11]^ In our case, APTT was prolonged, and bleeding was likely to occur. Furthermore, the prolonged APTT was caused by acute myocardial infarction and accompanying circulatory failure. The D-dimer level also increased, and platelet count decreased. Therefore, we considered the patient to have disseminated intravascular coagulation with increased fibrinolysis due to predominant coagulation. Consequently, general heparin control to prevent thrombosis was not possible. Considering these conditions, it is highly likely that VA-ECMO contributed to the spinal cord infarction.

Discontinuing sedatives and performing regular neurological examinations of the lower limbs are also important for early detection of spinal cord infarctions.^[12]^ However, even when the sedative was discontinued on day 3 as in our case, the patient’s consciousness remained impaired, and regular neurological examinations were not possible. However, a spinal cord infarction diagnosis was made after ECMO withdrawal in all the other reported cases except 1 (Table 1), emphasizing that early diagnosis is very challenging. Moreover, after spinal cord infarction onset, it is considered important to maintain a mean blood pressure of 90 mm Hg and increase spinal cord perfusion pressure,^[12]^ but these measures were either not possible or not described in the reported cases (Table 1).^[8,9,12–16]^ Similarly, we did not actively raise blood pressure so as to protect the heart after the myocardial infarction. Notably, patients with VA-ECMO often have hypocardiac function; therefore, maintaining an average blood pressure of 90 mm Hg may be difficult. Of the 20 cases presented in Table 1, the mean blood pressure was unable to maintained at 90 mm Hg in only 2 cases,^[12]^ including the present case. Both these patients were in cardiogenic shock and were unable to maintain a mean blood pressure of 90 mm Hg. In both cases, there was no improvement in the neurological findings. Although limited by the small number of cases, maintaining a mean blood pressure of 90 mm Hg appears to be difficult but important to raise spinal cord perfusion pressure and avoid neurological complications in the lower limbs in patients with VA-ECMO.

## 4. Conclusion

After VA-ECMO, spinal cord infarction is rare but possible, with a high probability of neuropathy. However, regular neurological examinations and high blood pressure maintenance, which are important tasks for early detection and damage prevention, are challenging in practice. Therefore, collection of more cases describing improvements after spinal cord infarction are needed to advance patient care.

## Author contributions

**Conceptualization:** Hideya Itagaki.

**Data curation:** Hideya Itagaki, Tomoya Oizumi, Keiko Nakagawa, Yoshinobu Abe.

**Formal analysis:** Hideya Itagaki.

**Funding acquisition:** Hideya Itagaki.

**Investigation:** Hideya Itagaki.

**Methodology:** Hideya Itagaki.

**Project administration:** Hideya Itagaki.

**Resources:** Hideya Itagaki.

**Software:** Hideya Itagaki.

**Supervision:** Hideya Itagaki.

**Validation:** Hideya Itagaki.

**Visualization:** Hideya Itagaki.

**Writing – original draft:** Hideya Itagaki, Kohei Suzuki.

**Writing – review & editing:** Hideya Itagaki, Tomoyuki Endo.

## Correction

Dr. Kohei Suzuki’s name has been corrected from Sizuki.
